# Structural, Synaptic, and Epigenetic Dynamics of Enduring Memories

**DOI:** 10.1155/2016/3425908

**Published:** 2016-01-06

**Authors:** Ossama Khalaf, Johannes Gräff

**Affiliations:** Laboratory of Neuroepigenetics, Brain Mind Institute, Faculty of Life Sciences, École Polytechnique Fédérale de Lausanne (EPFL), 1015 Lausanne, Switzerland

## Abstract

Our memories are the records of the experiences we gain in our everyday life. Over time, they slowly transform from an initially unstable state into a long-lasting form. Many studies have been investigating from different aspects how a memory could persist for sometimes up to decades. In this review, we highlight three of the greatly addressed mechanisms that play a central role for a given memory to endure: the allocation of the memory to a given neuronal population and what brain areas are recruited for its storage; the structural changes that underlie memory persistence; and finally the epigenetic control of gene expression that might regulate and support memory perseverance. Examining such key properties of a memory is essential towards a finer understanding of its capacity to last.

## 1. Introduction

**Figure 1:**
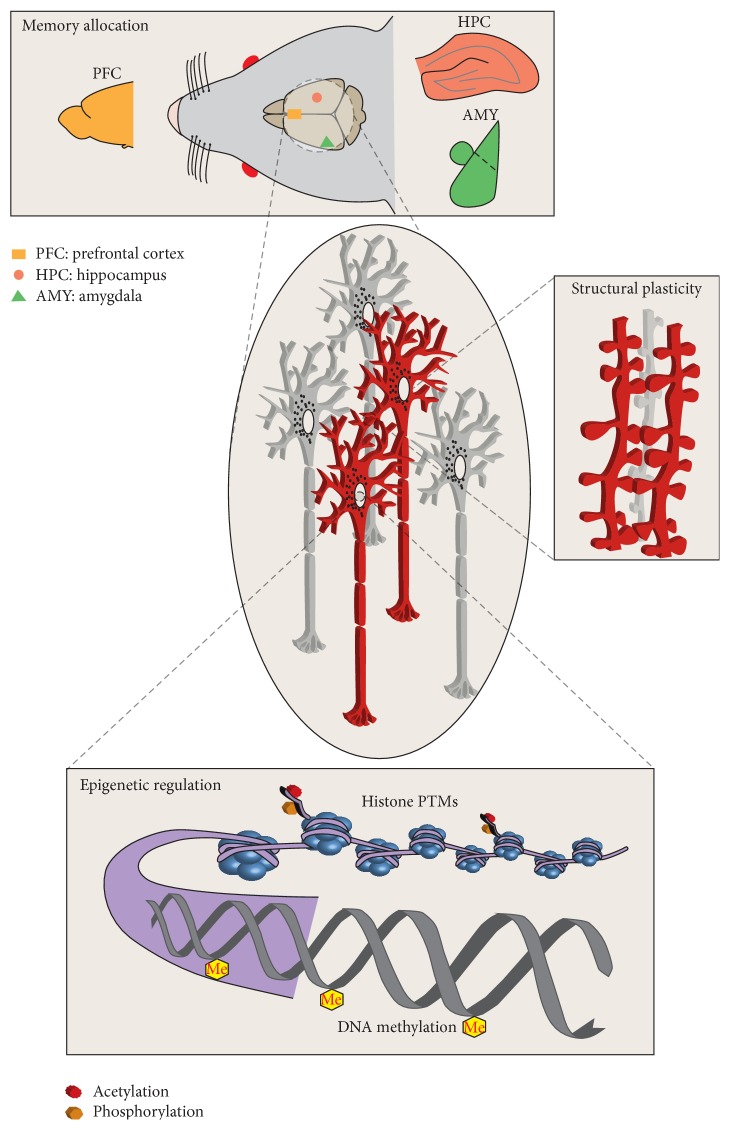
Schematic illustrating three essential mechanisms that might contribute to remote memory storage and thus memory endurance in the (rodent) brain, which are discussed in this review. First, during memory allocation, learning induces the activity of a specific subpopulation of cells—likely spread across different brain areas—which will become recruited into the memory trace. The amygdala (AMY), the hippocampus (HPC), and the prefrontal cortex (PFC) are known to be activated during memory allocation (for details see text). Second, in cells allocated to a specific memory—also known as the memory engram [1–3]—structural changes at the level of dendritic spines have been demonstrated by several studies. These changes are exclusive to the cells of the memory trace or engram (red) but not observed in other cells (grey) [53]. Third, memory engram cells are also likely to be characterized by epigenetic changes, such as posttranslational modifications (PTMs) on histone proteins, and methylation of the DNA, the core chromatin constituents. Note, however, that such engram-specific engagement of epigenetic mechanisms remains to be experimentally demonstrated.

Based on experience, memory is the capacity of an individual to acquire, store, and retrieve information. The physical substrate of such memories in our brains is known as memory trace or as first coined by the German biologist Semon (1859–1918) as “engram” [1–3]. One of the fundamental questions in memory research is how the experiences that we acquire transform into engrams that persist over time. It is generally acknowledged that the records we form from our daily experiences are not stored instantaneously but rather retained in an initially labile state that gradually transforms into a more stable trace or engram that is characterized by resistance to disruption [4–6]. Although this view has been challenged by the reconsolidation hypothesis, stipulating that even a stably stored memory could become transiently sensitive to disruption upon recall [6, 7], it is evident that not all forms of memories are amenable to disruption [8]. This is particularly relevant for strong memories, induced by an intensive training protocol, and long-lasting forms of memories, ranging from several weeks to months [9, 10] in age. Based on these grounds, but notwithstanding several studies testifying to the amenability of even long-term memories to disruption [11, 12], in this review we focus on 7-day-old—and older—memories as being remote and with the potential to endure, and we outline three mechanisms that might contribute to such endurance: first, memory allocation and storage; second, structural neuronal changes; and third, nuclear epigenetic dynamics (Figure 1).

Memory allocation refers to an early process by which certain neural circuits are assigned to stow a specific memory and what might favor the allocation of a memory into a specific population of neurons over others. In this review, we focus on some of the well-described elements that govern such allocation; still it is clear that we are only at the beginning of understanding the entire process of memory allocation, and many more aspects thereof remain to be identified. Once allocated, the question of where the memory is stored and what brain regions upkeep the memory is another one of utmost importance. The whereabouts of a specific memory is thought to be dependent on how old this memory is. The more nascent it is, the more it will be hippocampal-dependent, but as it matures it will change such dependence to higher cortical regions [13, 14]. Here, we describe brain areas that have been defined to be essential for the support of a long-lasting memory.

Furthermore, many neuroscientists believe that memories are encoded into neurons as structural changes in synaptic connections. Indeed, such structural plasticity is under comprehensive study in order to understand how brain circuits are modifying themselves in terms of number and strength of synaptic connections that correlate with the persistence of a memory [15–17]. We discuss these physical changes in synapses and their potential to support enduring memories.

Lastly, we also discuss the epigenetic modifications that are associated with long-lasting memories. We shed light on such modifications to the DNA or the histone tails that could lead to a cascade of changes in gene expression, a key feature of long-term memories [18], and which might thereby be able to assist memories to persist throughout the life of an individual.

## 2. Memory Allocation and Storage

Once formed, memories gradually transform from an initially vulnerable state to a more permanent state that is increasingly persistent to disruption. Such process of postexperience memory stabilization was first described by Müller and Pilzecker referring to it as “memory consolidation” [4, 5]. Later, two different types of memory consolidation have been distinguished: cellular/synaptic and systems consolidations. Cellular consolidation is a rather fast process taking place within the first few hours following learning and necessary for the initial stabilization of memories in hippocampal circuits [13]. In contrast, the systems consolidation process is slower and involves a time-dependent, gradual reorganization of the brain regions that support the memory, with the memory dependence shifting from the hippocampus to cortical regions [14]. This led to the contemporary view of systems consolidation which states that the hippocampus (HPC) is merely a temporary store for new information, while its permanent storage depends on largely distributed cortical networks [14].

In this section, we review what molecular and cellular events govern memory allocation in or to a certain neuronal population and then what brain regions support long-lasting memory storage.

### 2.1. Memory Allocation

By definition, memory allocation is the set of processes that determine where information is stored in a particular neural circuit [19]. Several studies showed that such allocation is not random but rather dependent on specific molecular mechanisms [20–22]. In one of these studies [20], using a viral vector Han et al. artificially increased the levels of CREB (cAMP responsive element-binding protein), a transcription factor important for the stability of synaptic potentiation and memory [23] in neurons of the lateral amygdala (LA), a subcortical brain structure implicated in emotional memories [24, 25], in mice. Twenty-four hours after a tone fear conditioning training, the mice were tested for the tone and sacrificed 5 min later. Using cellular compartment analysis of temporal activity by fluorescence* in situ* hybridization (catFISH), LA neurons transfected with CREB—identified by its GFP fluorescent tag—were found to be three times more likely than their neighboring nontransfected cells to express activity-regulated cytoskeletal (*Arc*), a gene required for synaptic function and memory [26, 27]. This suggests that CREB levels bias neurons to become part of the engram and to be encoded by the tone conditioning in the amygdala.

In a subsequent loss-of-function study, cells that were virally transfected with CREB in the same behavioral paradigm were ablated using diphtheria toxin receptor (DTR). In this system, the expression of the DTR is inducible by the Cre-recombinase, which is also found in the same viral construct, making all the cells that receive the construct eventually express the DTR. Following the tone test (24 h after training), the mice were injected with the diphtheria toxin (DT) that will only interact with the cells expressing the DTR and kill them. The experimental group (CREB viral vector transfected and DT injected) showed a significant impairment in tone conditioning when tested 2 days after the DT injection [21]. Similar results were obtained using a different approach that allows for reversible neuronal activation instead of permanently killing the cells [22]. There, the* Drosophila* allostatin inhibitory receptor was delivered to the LA through the same viral construct providing CREB, and pronounced amnesia for tone conditioning was obtained as a result of inactivating these cells by allostatin peptide treatment. This amnesia was reversed upon retesting the mice one day later without the allostatin peptides demonstrating the reversibility of the allostatin effects and the link between activity in the CREB cells and recall [22]. Despite the exclusive focus on CREB in the previous studies, the convergent findings using three different strategies strongly support its important role in memory allocation in the amygdala.

Another influential factor that determines the allocation process appears to be neurogenesis in the dentate gyrus (DG). Using 5-bromo-2′-deoxyuridine (BrdU), a permanent stain that intercalates with dividing DNA allowing the tracing of newly born neurons, a recent study showed that 4- to 8-week-old DG neurons are preferentially recruited after spatial learning [28]. In contrast, 2-week-old neurons integrated with lower efficiency and 1-week-old neurons did not integrate at all [28]. In line with a recent study showing that 4-week-old (but not 1-week-old) neurons have the essential synaptic structure and physiology to support the appropriate connections with hippocampal circuits [29], this suggests that the timing of neuronal development relative to training is indeed vital in the memory allocation process. Nevertheless, the nature of memory allocation processes that take place in brain areas devoid of neurogenesis and outside the amygdala remains to be determined.

### 2.2. Memory Storage

After the initial allocation of a memory to a specific neural circuit begins the more prolonged process of systems consolidation that involves gradual reorganization of the brain regions that support memory formation and storage [13, 14]. Classical studies characterizing memory loss in patients with lesions of the medial temporal lobe (MTL) [30, 31] revealed that the hippocampus serves as a temporary store for new information, but that permanent information storage depends on a broadly distributed cortical network [14]. These human data are indeed consistent with observations that hippocampal lesions in the first week after training, but not thereafter, disrupt contextual fear memories in rats, and thus, maintaining a proper hippocampal trace is crucial to establish remote memories in the cortex [32]. From more refined studies, several molecules have in the meantime been identified that maintain the hippocampal trace of a memory in the days following training for the persistence into a remote memory [33, 34] (for a more detailed overview of other molecules that are involved in memory storage, but that have not been specifically assessed for remote memory storage, the reader is referred to [19]). For instance, when NMDA (*N*-methyl-D-aspartate) receptor (NMDAR) function was inducibly suppressed in the CA1 region in the week following the training of two hippocampal-dependent tasks (Morris Water Maze and contextual fear conditioning), remote memory formation for these tasks was blocked. However, when done at later time points, the suppression of the NMDAR function did not affect the remote memory formation [33]. Similar results were obtained when levels of *α*-calcium/calmodulin kinase II (*α*-CaMKII), a signaling enzyme mainly expressed in the excitatory neurons of the forebrain and essential for neuronal plasticity [35], were altered [34]: overexpressing a dominant-negative form of *α*-CaMKII in the week after training, but not afterwards, blocked the formation of remote contextual fear memories [34]. Together, these results support the importance of the HPC, especially during the first week following encoding, for memory consolidation in cortical networks and furthermore suggest that there is a crucial week-long window during which normal hippocampal activity is needed for the memory to be consolidated.

However, several studies found that cortical regions are also implicated in the initial phase of memory formation [36–39], thus challenging the idea that the HPC is solely involved in this process. In one of the recent studies in this regard [38], real-time optogenetic inhibition of excitatory medial prefrontal cortex (mPFC) neurons during contextual fear conditioning showed that such temporally precise inhibition impaired the formation of long-term associative memory, tested 30 d after of acquisition [38]. In another recent study [39], using a doxycycline-inducible mouse line (TetTag) to tag the activated neurons [40], optogenetic stimulation of the activated neural population during contextual fear memory training in the retrosplenial cortex (RSC), a cortical region implicated in episodic memories and emotional associations [41–44], was sufficient to produce fear memory retrieval even when tested until 2 d after acquisition [39]. These results are in line with previous studies [36, 37] showing that the PFC is critically involved in memory encoding and that its inactivation by local infusion of NMDAR antagonist could block contextual memory acquisition in mice [36] and learning of new paired-associates in rats [37].

In another intriguing study, Lesburguères et al. used a social transmission of food preference (STFP) test, where an associative olfactory memory develops after a study animal (observer) learns about the safety of a certain food (novel odor for the observer) from an interaction session with another animal that has already tasted the food (demonstrator). Then the observer shows reduced fear towards this novel food upon the first encounter and significant consumption thereof. The authors first showed that the acquisition of such food preference memory is dependent on the orbitofrontal cortex (OFC) only for 30-day-old remote memory, but not for recent memory (24 h after training), and that for the first period after training (7 d) it is mainly HPC-dependent [45]. Nevertheless, the authors then went on to show that there is an intricate interplay between the HPC and the OFC for such memory to endure. Using the excitatory glutamate receptor antagonist 6-cyano-7-nitroquinoxaline-2,3-dione (CNQX) to block the activity of the OFC during the 2-week period following training, an unexpected memory loss to a novel odor test was observed 30 d later. Likewise, inactivating the OFC immediately before training blocked the memory after 30 d, and not after 7 d, indicating that early cortical activity is required for subsequent stabilization of such memory [45].

Beyond memory formation, several studies investigated the role of extrahippocampal structures in remote memory storage, from which the anterior cingulate cortex (ACC) emerges to play a key role at least in remote contextual fear memory storage [46–49]. Thus, lidocaine-mediated pharmacological inactivation of the ACC disrupts the retrieval of remote contextual fear memory in mice 18 d and 36 d after training, while inactivating the prelimbic cortex (PL)—a region located near the ACC in the mPFC—at the same time points did not disrupt the very same memory [46]. Similarly, the lidocaine-mediated inactivation of the PFC and the ACC was shown to impair remote spatial memory retrieval when tested 30 d after acquisition [47]. These results are in line with previously reported data from a study using noninvasive functional brain imaging to examine the metabolic activity of different brain regions underlying spatial discrimination memory storage in mice [48]. In this study, increased metabolic activation in the frontal cortex, together with the recruitment of the ACC and temporal cortices, was observed 25 d—but not 5 d—after acquisition [48]. Together, these findings indicate a high level of involvement of cortical areas during the retrieval of remote memories, postulating these areas to be vital structures for remote memory storage.

Finally, from a reconsolidation point of view and how memory storage could affect such process, it has been previously demonstrated that infusing anisomycin (ANI), a protein synthesis inhibitor, to the dorsal HPC (dHPC) or the ACC after contextual fear memory recall (45 d or 30 d after acquisition, resp.) disrupts the memory when tested 1 d after anisomycin treatment [11, 49]. Collectively, these results highlight an equal importance of hippocampal and cortical regions in remote memory reconsolidation, which suggest that probably the process of memory formation and storage does not depend solely on a single brain area but is more distributed among different structures that share the upkeep of the trace.

## 3. Structural Changes

Amongst many aspects that categorize a memory to be remote is persistence, yet how this property is achieved is still enigmatic. The strength and number of synaptic connections that are formed after an experience offer one possible explanation as to how remote memories could endure and last throughout life [18]—since we know that such processes—such as increased dendritic spine density—are indeed implicated in 1-day-old memories [15, 50, 51]. In this section, we shed light on the structural changes that modify the connectivity of brain networks and that might underlie remote memory perseverance.

A few years ago, Restivo and colleagues used contextual fear conditioning as a behavioral paradigm to show that recent and remote memory formation trigger region-specific and time-dependent morphological changes in hippocampal and cortical networks of mice [16]. Right after fear conditioning, there was a significant increase in spine density in the CA1 field of the hippocampus compared to the naïve or even pseudoconditioned groups. 36 days later, in contrast, this increase in spine density had developed sequentially when it reached the cortical regions, specifically the ACC. Thus, hippocampal plasticity* per se* is seemingly crucial in driving the structural changes that were observed at a remote time point, yet its role was merely time limited, an observation that was recently confirmed using time-lapse two-photon microendoscopy [52]. To further prove this assumption, a hippocampal lesion was generated early at the day of conditioning, where it abolished the growth of significant spine density in the ACC (36 d after training) compared to the sham group [16]. In contrast, when this lesion was introduced at a later time point (24 days after conditioning), it did not prevent the spine density changes in the ACC neurons. The detected structural changes in either region were directly correlated to the strength of the conditioned memory: an absence of these structural changes in the hippocampal or the cortical regions was accompanied by memory impairments for recent and remote memories, respectively. This is in line with a recent demonstration that such increase in synaptic density and plasticity occurs exclusively in engram cells, but not in nonengram cells, in the DG 24 h after encoding [53].

Importantly, such structural remodeling in hippocampal and cortical regions is essential for memory stabilization and afterwards for remote memory expression. The spine growth at the hippocampal neurons is important at an early time point after conditioning, yet this importance starts to fade with time, when a more permanent trace is formed in the cortex [17], as illustrated by the following study. To inhibit the structural changes that occur in the cortex, a transcription factor that is known to negatively regulate spine growth, myocyte enhancer factor 2 (MEF2), was overexpressed through a viral vector to increase the MEF2-dependent transcription in ACC neurons at 2 different time points, either 1 day or 42 days after conditioning. At the earlier time point, the stabilization of the conditioned memory and the associated increase in spine growth was blocked, whereas no effect was observed at the later time point [17]. This suggests that the increase in spine growth at the ACC following conditioning happens in a time-dependent manner and that it is central for the stabilization and persistence of such memory.

In contrast to the abovementioned studies, another study showed a rapid formation of new spines in the motor cortex of mice following a novel motor skill learning task [54]. Using* in vivo* superficial dendrites imaging, they demonstrated that there is an immediate formation of spines in the motor cortex following a novel motor learning task (within 1 h after learning initiation) and that these spines are preferentially stabilized upon subsequent training and endure long after training stops (up to 120 d) [54]. This suggests that the early cortical structural changes during motor learning and the subsequent stabilization over months subserve as long-lasting structural basis for memory maintenance and persistence of a motor skill. Similarly, a more recent study reported that the encoding of a long-term episodic memory itself elicits early structural changes in neocortical regions. In this study, structural plasticity in the mPFC was significantly increased 1 h following contextual fear conditioning [38]: investigating the morphology of individual dendritic spines on mPFC pyramidal neurons revealed that the ratio of the thin spines to mushroom spines was significantly increased following conditioning. This suggests that dendritic spine plasticity in the mPFC circuit also contributes to memory encoding, which is surprising as the remodeling of the cortex was traditionally thought to be limited to the later stages of memory processing that promote remote memory storage [55]. Further investigations are now needed to have a better understanding of these structural changes and how they are employed to serve memory lasting or extinction (Box 1).

## 4. Epigenetic Regulation

Remote memories persist throughout the life of individuals, whereas the protein molecules that may subserve these memory traces are thought to turn over on the order of days [56]. To address such unanswered questions dealing with the molecular basis for a lifelong memory, it has been proposed by Crick (1916–2004) in 1984 and later on by the molecular biologist Holliday (1932–2014) in 1999 that epigenetic mechanisms—particularly DNA methylation—could partly explain the persistence of memories over a lifetime [57, 58]. Epigenetics has long been heralded as a stable and self-perpetuating regulator of cellular identity through establishing persistent and heritable changes in gene expression across cell divisions [20]. Although the nervous system is essentially composed of nondividing cells, the recent decade has shown that epigenetic mechanisms could nevertheless play a fundamental role in forming lasting memories.

Commonly, DNA is packaged into chromatin through its wrapping around octamers of histone proteins. Chromatin can exist either as heterochromatin or as euchromatin: heterochromatin is characterized by condensed chromatin and subsequent transcriptional repression, whereas euchromatin is characterized by a relaxed chromatin state that allows the transcriptional machinery to access the DNA for gene expression [59]. Apart from short interfering RNA molecules that mediate posttranscriptional gene silencing [60] and induce epigenetic changes in gene expression via modifications of chromatin [61], the switch between both states of chromatin is governed by two major epigenetic modifications: DNA methylation and posttranslational modifications (PTMs) on histone tails. DNA methylation refers to the covalent addition of a methyl group to the cytosine base by DNA methyltransferases (DNMTs), while PTMs are the addition and removal of chemical moieties to histone tails, which are dynamically regulated by chromatin-modifying enzymes [22]. These modifications include—but are not limited to—histone acetylation, phosphorylation, and methylation [62] (see Tweedie-Cullen et al., for a complete overview of recently identified PTMs in the brain [63]). Both types of epigenetic modifications are associated with learning and memory, and many recent studies have shown that these epigenetic changes could support memory formation and maintenance through a cascade of specific changes to gene expression including enduring memories.

### 4.1. DNA Methylation

The first study to investigate the potential role of DNA methylation in regulating memory formation by Sweatt and colleagues showed that* Dnmt* gene expression is upregulated in the adult rat hippocampus following contextual fear conditioning and that its inhibition blocks memory formation [64]. Accordingly, fear conditioning was associated with an upregulation of mRNA levels of the DNMT subtypes that are responsible for* de novo* methylation, DNMT3A and DNMT3B, in the CA1 region 30 min after training. Then, to show that the hippocampal DNMT activity is necessary for memory consolidation, DNMT inhibitors—5-azadeoxycytidine (5-AZA) or zebularine (zeb)—were locally infused right after the training, where they abolished the freezing response of the injected group 24 h after (test day 1). Interestingly, when retrained immediately after test day 1 and retested 24 h later (test day 2), the DNMT inhibitor-treated group showed significantly higher freezing than on test day 1, and when retrained and retested 24 h later (test day 3), they showed equivalent freezing to the vehicle-treated group. But when 5-AZA was infused 6 h after training and animals were tested 18 h later (24 h after training), the inhibitor-injected group displayed normal fear memory indicating that the effect of DNMT inhibition is merely due to blocking consolidation and not due to any other effects on the retrieval or the performance of the animals [64]. These experiments suggest that the transient inhibition of DNMT in the hippocampus following training blocks memory consolidation in a resilient manner that could be reverted as soon as the inhibitor clears off and that the necessary DNA methylation states for consolidation could be reestablished.

In a follow-up study, Miller et al. found a rapid increase in methylation of a memory-suppressor gene in the hippocampal CA1 region 1 h after contextual fear conditioning. Using quantitative real-time PCR, the methylation levels of protein phosphatase 1 (*PP1*), a memory-suppressor gene that is suggested to promote memory decline [65], were dramatically higher in the fear-conditioned group compared to the control group. This increase in methylation was associated with lower levels of* PP1* mRNA, yet the increase in methylation was attenuated and associated with a twofold increase in the mRNA levels when 5-AZA was infused locally 1 h after training. Conversely, a demethylation of a memory-promoting gene was found in the CA1 region 1 h after contextual fear conditioning. The demethylation of* reelin*, a gene that enhances long-term potentiation and the loss of function of which results in memory formation deficits [66, 67], was pronounced in the trained group with its mRNA levels being significantly higher than the control group. DNMT inhibition using 5-AZA led to further demethylation of* reelin* and even higher levels of its mRNA. These data suggest that the DNA methylation is dynamically regulated and that it is a crucial step in memory formation.

Importantly, cortical DNA methylation also seems to support remote forms of memories [68]. The cortical DNA methylation of the memory-suppressor* calcineurin* (*CaN*, also known as* Ppp3ca*), a gene that downregulates pathways supporting synaptic plasticity and memory storage, was investigated using methylated DNA immunoprecipitation (MeDIP) in rats.* CaN*'s cortical DNA methylation persisted for at least 30 d after contextual fear conditioning, and its mRNA levels were significantly reduced in the trained group 2 h after retrieval 30 d after training. Importantly, when the NMDA receptor antagonist (AP5) was infused into the dorsal hippocampus (CA1) just before training,* CaN* methylation in the dorsal medial prefrontal cortex (dmPFC) 7 d after training was blocked, indicating that a single hippocampus-dependent learning experience is sufficient to drive lasting, gene-specific methylation changes in the cortex. Moreover, intra-ACC infusions of DNMT inhibitors (5-AZA or zeb or RG108) 30 d after training disrupted fear memory and were associated by a significant reduction in the* CaN* methylation levels. However, the infusion of these inhibitors 1 d after training had no effect on fear memory 30 d later [68]. These results indicate that cortical DNA methylation is indeed triggered by a learning experience, and most importantly, its perpetuation supports long-lasting, persistent memories. More detailed studies including investigating DNA methylation changes on a genome-wide scale or within engram-bearing cells are clearly warranted to deepen our knowledge of the implication of these changes in remote memory storage.

### 4.2. Histone PTMs

Newly formed hippocampus-dependent memories need to be stabilized into a long-lasting ACC-dependent memory trace [46, 69, 70]. Several studies demonstrated that changes in gene expression in both brain regions accompany such stabilization [46, 47]. This differential gene expression has recently been associated with epigenetic modifications in terms of histone PTMs [71]. Using a novel object recognition task on mice, serine (S) 10 phosphorylation on histone (H) 3, lysine (K) 14 acetylation on H3 as well as H4K5 acetylation, and H3K36 trimethylation in the PFC associated with remote (7 d after training) memory consolidation. Importantly, the doxycycline-inducible selective inhibition of the memory-suppressor gene* PP1* in a transgenic mouse line showed improved remote memory performance accompanied by increased histone PTMs. In contrast, blocking the occurrence of these PTMs using a cocktail of inhibitors targeting the epigenetic enzymes responsible thereof impaired remote object memory, suggesting that these histone PTMs are essential for memory consolidation and retention. Finally, these histone PTMs were increased in the promoter region of* Zif268*—an immediate early gene important for memory formation and storage [72]—and its expression levels shift from the hippocampus to the PFC as the memory matures [71]. This study shed light on the spatiotemporal dynamics of these histone PTMs in the hippocampus and cortex and demonstrated that they could act as molecular marks subserving memory consolidation—at least up to 7 d after training.

Similar results were obtained for memory consolidation of social transmission of food preferences [45]. There, associative olfactory memory was linked to a marked increase in H3 acetylation in the OFC 1 h after training, but such increase disappeared upon inactivating the OFC using tetrodotoxin or CNQX. Additionally, increasing the OFC histone acetylation by infusing HDAC inhibitors (sodium butyrate or trichostatin A) was associated by an increase in memory robustness at the remote time point (30 d) [45]. Together, these results stipulate that this cortical epigenetic mark observed very early during training might be essential for tagging these neurons to allocating them to the long-term olfactory memory and that thereafter these neurons will participate in the system consolidation process driven by the HPC-OFC circuitry in order to help this memory to endure. It would be highly interesting to repeat this study with CREB-transfected OFC neurons in order to test this hypothesis.

In addition to histone PTMs, a recent study by Zovkic et al. has shown that a variant of histone H2A (H2A.Z) is actively exchanged in the hippocampus and cortex in response to fear conditioning in mice [73]. H2A.Z is known to be associated with nucleosomes adjacent to the transcription start site (TSS) of a gene, and its presence has been strongly linked to dynamic changes in gene expression [74]. To investigate its effect on transcriptional changes associated with learning, chromatin immunoprecipitation (ChIP) was used. Binding of H2A.Z was reduced at the +1 nucleosome (first nucleosome downstream of the TSS) of memory-promoting genes (*Npas4*,* Arc*,* Egr1*,* Egr2*, and* Fos*), and there was an increase in the expression of those genes 30 min after the contextual fear training. In contrast, H2A.Z binding was increased for the memory-suppressor gene* CaN* and associated with reduced expression of this gene. This suggests that H2A.Z at the +1 nucleosome restricts memory-related gene transcription [73]. Furthermore, the methylation of the promoter region of the gene encoding H2A.Z (*H2afz*) was shown by MeDIP to be increased 30 min after contextual fear conditioning, when it was accompanied by reduced H2A.Z protein expression throughout the hippocampus, whereas the expression levels of H2A.Z returned to baseline after 2 h [73].

To assess a causal involvement of H2A.Z in memory consolidation, an adenoassociated virus (AAV) depleting H2A.Z in the dorsal CA1 region of the hippocampus was used. This approach improved fear memory 24 h and 30 d after training compared to a scramble-injected control group. In contrast, when H2A.Z was depleted from the mPFC, there was no effect on fear memory at the hippocampus-dependent 24 h time point, yet the freezing was significantly higher at remote time points 7 and 30 days after training [73]. Moreover, a genome-wide transcriptional analysis was carried out to evaluate the impact of H2A.Z depletion on training-induced gene expression in CA1 and mPFC 30 min after training. The analysis showed a differential expression—between the trained and untrained groups—in many genes including a number of the early learning-related genes:* Arc*,* Fos*,* Egr1*, and* Egr2* [73]. Although the study did not ascertain the specific target genes through which H2A.Z regulates memory, it clearly demonstrated that H2A.Z is dynamically regulated during learning and memory and that it could be an important epigenetic contributor to the complex coordination of gene expression in memory. Future, more refined studies will certainly help to elucidate the role of histone exchange and histone PTM processes associated with remote memory storage or extinction (Box 2).

## 5. Summary

The allocation of a memory to a particular neural circuit is a critical step in memory formation. We reviewed how CREB is involved in such process highlighting its important role. Additionally, electrophysiological studies showed that cells transfected with CREB viral vectors are more excitable compared to the neighboring cells or even those transfected with the control vector [22]. This could partially address the preference of allocating the memory to CREB cells since their increased excitability might render them more responsive to sensory inputs and therefore more likely to get activated during conditioning training. However, it could still be possible that there are other molecular determinants and processes that are important for memory allocation. Indeed, although CREB is ubiquitously expressed, it seems unlikely that memory allocation depends solely on this transcription factor. Likewise, adult neurogenesis is restricted to only certain brain regions, and the data showing that new granule cells when mature are increasingly likely to be incorporated into circuits supporting spatial memory [28, 29] is not necessarily the sole determinant of allocating a memory to a specific neural population.

Another important aspect of memory persistence is which brain regions maintain its storage and what supports such perseverance. We highlighted the importance of the ACC in the upkeeping of remote memories since its inactivation prevents the recall of remote contextual fear memory as well as the reconsolidation of such remote memory 24 h after its retrieval [46, 49]. Intriguingly, a recent study identified for the first time monosynaptic projections from the ACC to the hippocampal CA fields that controls memory retrieval in mice [75]. Using retrograde tracers, this study characterized novel connections between ACC and CA fields (AC-CA) that subserve a potential bidirectional communication between the ACC and the hippocampus. Manipulating these projections optogenetically demonstrated a causal top-down control on memory retrieval, where the cells contributing to the AC-CA projection can activate contextually conditioned fear behavior (3-day-old memory), whereas their inhibition impaired the retrieval of such memory [75]. Nevertheless, further investigations are still needed to elucidate the role of these projections on the regulation of different memory processes.

In fact, the cellular reconsolidation of a remote memory might not solely depend on the ACC since it has been shown previously that infusing anisomycin in the dHPC blocks the reconsolidation of remote contextual fear memory and that optogenetically inactivating the CA1 region would even impair recalling it [12]. Contradictorily, another study did not find any evidence that neither the ACC nor the dHPC is involved in the cellular reconsolidation of remote contextual fear memory following retrieval [76]. More studies are highly anticipated to resolve these divergent findings, although such discrepancy could be partly attributed to the difference in the strength and length of the training and retrieval sessions used or in the inactivation method and its efficiency, since it has been demonstrated that these experimental conditions significantly affect the behavioral outcome [10, 77].

Structural plasticity is another key point towards understanding the endurance of some memories. It provides a physical substrate for the storage of memories. We highlighted the synaptic plasticity that follows memory formation at hippocampal dendrites and that such plasticity reaches cortical areas in a time-dependent manner [16, 17]. Nonetheless, we also shed light on two interesting studies supporting the view of an early cortical reorganization during motor skill learning [54] as well as episodic memory acquisition [38], which demonstrated the importance of such structural changes for lasting memories. The reduced density of spines in cortical areas upon remote fear extinction is in line with these findings and suggests remodeling in the cortical circuit of the original memory [78]. However, a contradicting study showed that it is rather fear memory formation that is accompanied by spine elimination and that extinction involves spine formation [79]. These results are quite confusing, and although they could also be reflecting that opposite processes are at play in different cortical areas, they need to be addressed properly soon.

The epigenetic regulation was the final point we highlighted in this review, and the data we reviewed—collectively—support a dynamic pattern of epigenetic modifications including both DNA methylation [68] and histone PTMs [71] that subserve a spatiotemporal shift of the memory trace from the HPC to higher cortical regions during the process of memory consolidation. Also, the early tagging of certain neurons with epigenetic marks during encoding is central for the memory to be allocated to the tagged neurons and for the subsequent participation of these neurons in the circuit supporting such memory [45]. Furthermore, the extinction of remote fear memories with an HDAC2i increased histone acetylation-mediated neuroplasticity [80], and the lack of such plasticity from the hippocampus upon remote memory recall supports the idea of hippocampal disengagement for remote memories [46, 48, 55]. Nevertheless, whether memories might indeed be “coded in particular stretches of chromosomal DNA” as originally proposed by Crick [57] and if so what the enzymatic machinery behind such changes might be remain unclear. In this regard, cell population-specific studies are highly warranted.

Taken together, we find ourselves in an exciting period witnessing an increasing number of studies, which dare to investigate remote memory formation, storage, and persistence. Yet it is clear that we are still in need of further investigations to unveil the dynamics of neuronal circuits and molecular mechanisms mediating such persistence. Ultimately, deciphering these processes would definitely contribute to the understanding, and possibly dulling, of abnormally long-lasting fear memories like those underlying anxiety disorders or posttraumatic stress disorder.

## Figures and Tables

**Box 1:**
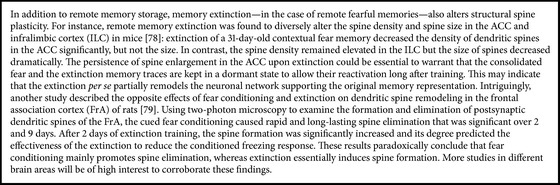
Recent insights into structural plasticity and remote fear memory extinction.

**Box 2:**
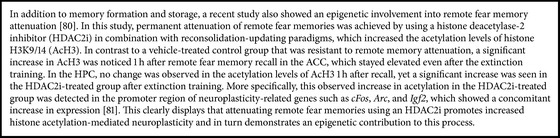
Recent insights into epigenetic dynamics of remote memory attenuation.
